# Access to oral health care for children with fetal alcohol spectrum disorder: a cross-sectional study

**DOI:** 10.1186/s12903-022-02561-z

**Published:** 2022-11-16

**Authors:** Katie Hu, Keith Da Silva

**Affiliations:** College of Dentistry, University of Saskatchewan, 123-105 Wiggins Road, Saskatoon, SK S7N5E4 Canada

**Keywords:** Fetal Alcohol Spectrum Disorders, Developmental Disabilities, Health Services Accessibility

## Abstract

**Background:**

Individuals with developmental disabilities, including Fetal Alcohol Spectrum Disorder (FASD), often suffer from poorer oral health than the general population as they experience challenges with accessing care. However, few studies have investigated access to oral health care specific to children diagnosed with FASD. Thus, the objective of this cross-sectional study is to examine the use of oral health care services by children diagnosed with FASD in Saskatchewan, Canada, and to identify perceived barriers that affect their access to oral health care.

**Methods:**

Parents or caregivers for children with FASD under the age of 16 were recruited through community organizations. Between July 2020 and January 2021, 189 participants completed a 64-item questionnaire that assessed sociodemographic characteristics, oral health care utilization, and perceived barriers to care.

**Results:**

Most children (85%) had visited the dentist within the last 24 months. 55% of children had required sedation for some treatment. 43% of caregivers experienced frustration trying to access care for their child. Common barriers were cost (63%), location (55%), the child’s behaviour (78%) and caregiver anxiety (60%). 35% of caregivers believed their dentist lacked adequate knowledge of FASD. Univariate analysis reveals that income, caregiver education, residence location, and insurance status were significantly associated with reporting barriers. Multivariate logistic regression analysis reveals that caregivers who reported a high school education (OR=1.23; 95% CI 1.03 – 1.38); or public insurance (OR=1.33; 95% CI 1.24 – 1.42) or out-of-pocket payments (OR=1.37, 95% CI 1.20 – 1.46); or rural (OR=1.19, 95% CI 1.07 – 1.26) or remote (OR=1.23; 95% CI=1.12 – 1.31) residences were more likely to report difficulties accessing oral health care.

**Conclusion:**

Our findings indicate that children with FASD experience various barriers to accessing oral health care. Social determinants of health were significant variables that increased likelihood of barriers. Like other vulnerable populations, cost and clinic location are notable barriers. Oral health care providers’ assessment and management of children with FASD are noteworthy for future research.

## Background

Individuals with special health care needs often face considerable barriers to accessing dental care [1, 2]. This is particularly true for children with developmental disabilities who can experience higher rates of untreated dental caries when compared to children without any disabilities [3, 4]. The oral health care needs of children with developmental disabilities are complex, and additional caries risk factors can include congenital anomalies, associated comorbidities, responsive behaviour, a need for specialized care, as well as an inability to maintain optimal oral hygiene [3, 5]. As such, understanding the unique oral health care needs of this population, as well as identifying any potential barriers to accessing care is of utmost importance.

One such developmental disability is Fetal Alcohol Spectrum Disorder (FASD). FASD is a diagnostic term used to describe impacts on the brain and body of individuals prenatally exposed to alcohol. It is estimated that 4% of Canadians are diagnosed with FASD, which is more than all other developmental disabilities combined [6]. Individuals diagnosed with FASD may have general growth impairments as well as defects in various organs including the brain, heart, kidneys, and bones [7, 8]. Sentinel facial features including short palpebral fissures, smooth philtrum, and thin upper lip may be present in children with FASD, as well as other facial or dental anomalies such as micrognathia, cleft lip and/or palate, small teeth with defective enamel, malocclusions, and delayed tooth eruption [7, 8]. Additionally, FASD is a complex neurodevelopmental disorder that can result in permanent functional disorders, including impaired cognition, behavior, intellectual functioning, attention, learning, and executive function [7, 9] Secondary adverse outcomes include chronic health issues, social adaptive dysfunction, delinquency, legal troubles, confinement and increased needs for services and supports [9, 10]. Specially for children diagnosed with FASD, many end up in the foster care system due to circumstances beyond their control [7].

Common social and behavioral challenges in individuals with FASD may include hyperactivity, social skills deficits, poor adaptive functioning, and externalizing behaviors such as rule breaking and aggression [10]. These challenging behaviors are often misinterpreted and misdiagnosed, especially in individuals without cardinal features, thus FASD is often referred to as hidden disability. This lack of awareness can increase the stigma associated with individuals with FASD as well as elevate the stress load on their caregivers [10, 11]. Caregivers often feel stigmatized and isolated from the community as they may be directly or indirectly blamed for their child’s behaviors [10–12]. Even those with knowledge of the child’s diagnosis, such as health care providers, may marginalize and blame birth parents for their contribution through prenatal alcohol exposure [10]. When considering medical care, the overarching construct identified for barriers to accessing care was a lack of health care provider knowledge of FASD, which can create bias and lead to delayed diagnosis or misdiagnosis as well as inadequate care [6, 10, 12]. Other barriers cited in the literature include insufficient caregiver knowledge as well as caregiver incapacity and stress [10].

Access to care involves the opportunity to obtain timely health care services when needed [13]. However, there are many barriers that impede the ability to access oral health care for individuals with disabilities which may be classified as external (environmental), internal or interpersonal [13, 14]. External environmental factors can include social determinants of health, the cost of care, employment status, structural barriers, transportation difficulties, and inadequate facilities [14, 15]. Internal factors to the patient and their caregivers may be medical, physical, cognitive, communication and/or behavioral issues [14, 15]. Lastly, interpersonal factors refer to the relationships between dental staff, patients, and caregivers.

While the oral health status and barriers to dental care for children with developmental disabilities such as autism has been explored [2–5], there is limited research available as it relates to children diagnosed with FASD. A recent retrospective study demonstrated that children with FASD living in Saskatchewan are at a higher risk for poor oral health outcomes, have more extensive treatment needs, and are at a higher risk for dental treatment under general anesthesia [16]. One potential explanation for these heightened risks could relate to challenges with accessing care. While there has been research that considers access to general health care services for individuals with FASD [11, 12], there has yet to be any research specific to oral health care. Thus, the objectives of this study are to examine the use of oral health care services for children with FASD living in Saskatchewan as well as to describe parental/caregiver perceptions on accessing oral health care for their child.

## Methods

This cross-sectional quantitative study is compliant with the STROBE checklist for cross-sectional studies and was approved by the University of Saskatchewan Behavioral Research Ethics Board (REB ID #1502). Parents and/or caregivers for children with FASD under the age of 16 were recruited through the FASD Network of Saskatchewan which serves approximately 400 clients. Using the rough national prevalence estimate of 4%, along with 95% confidence interval and 3% sample error, our sample size calculation estimated that a minimum of 96 participants were required for this study. Participant recruitment and data collection occurred between July 2020 and January 2021.

A 64-item questionnaire was developed based on a framework for access to care (Levesque) and considered external (environmental), internal, and interpersonal barriers to accessing care. The questionnaire assessed basic demographic and socioeconomic characteristics (family income, education level, residence location etc.), oral health care utilization (services, visits, provider type etc.), and perceived barriers to care (location, cost, anxiety etc.). A Likert type scale was used for the questions regarding barriers, where the “occasionally” and “sometimes” responses were combined into one variable. After pilot-testing and refining the survey questions, an electronic questionnaire (SurveyMonkey®) was distributed through social media and email. Each participant was guided through the informed consent process and consent was recorded prior to starting the questionnaire. All participants enrolled in the study received an honorarium for their time.

Questionnaire responses were cleaned, coded, and entered to Microsoft Excel. A quality check using a random sample of surveys was conducted to check for potential data entry errors. To describe our study population, we used frequencies and absolute numbers for categorical variables, and means and standard deviations for continuous variables. To first explore the association between a caregivers reporting difficulty accessing oral health care and selected sociodemographic variables, a univariate analysis was used where these categorical variables were individually assessed using Chi-squared tests for independence. When one or more of the sample data points in a set was less than five, Fisher exact tests were used. Next, to determine which factors were independently associated with accessing oral health care, we created a multivariable logistic regression model with the outcome being reporting difficulty accessing oral health care. Variables with a P-value less than 0.05 as determined in the univariable analysis were included as independent variables, and assessed in terms of odds ratio (OR) and 95% confidence intervals (95% CI). The significance level was set at 0.05. All data analyses were performed using Statistical Package for the Social Sciences (SPSS) v.23.

## Results

A total of 189 parents or caregivers of children with FASD participated in this study for an estimated response rate of 47%. Basic demographic data is presented in Table 1. Almost two-thirds of caregivers (60%) were directly related to the patient (biological parent or relative), while roughly 40% were either adoptive or foster parents. Approximately 15% of caregivers had less than high school education, and 57% reported an annually family income of less than $50,000. The geographic distribution of primary residence varied, with 46% of caregivers living urban centers, 31% living in rural areas (less than 2 hours’ drive from a city) and 23% living in more remote locations (more than 2 hours’ drive from a city). Regarding dental insurance status, most of the sample population (61%) relied on some form of publicly funded insurance.

**Table 1:** Participant demographics and characteristics, n (%) or mean ± SD

Total Sample	*n* =189
Child’s age (years)	10.61 ± 1.03
Child’s gender	
Male	102 (54)
Female	87 (46)
Caregiver status	
Biological parent	59 (31.4)
Relative	55 (28.9)
Adoptive parent	67 (35.5)
Foster parent	8 (4.1)
Caregiver highest education	
Less than high school	29 (15.3)
High school or equivalent	57 (30.2)
Post-secondary (college, university)	67 (35.4)
Post-graduate/Doctoral	36 (19.1)
Family income	
Less than $20,000	28 (14.9)
$20,000 to $49,999	80 (42.3)
$50,000 to $74,999	63 (33.3)
Greater than $75,000	18 (9.5)
Insurance status	
Private insurance	62 (33.3)
Public insurance	116 (61.4)
Out-of-pocket payments	11 (5.8)
Primary residence	
Urban	108 (57.1)
Rural (< 2 hours from a city)	58 (30.7)
Remote (> 2 hours from a city)	23 (12.2)

A summary of oral health care behaviour and utilization is presented in Table 2. The majority of children (85%) had visited the dentist within the last 24 months, with dental examinations (95%), radiographs (90%), fillings (80%), and extractions (68%) being the most frequently reported services. Approximately 55% of caregivers reported that their child required either sedation or general anesthesia for dental treatment on at least one occasion. Almost 75% of caregivers reported their child brushes at least once per day; however, 68% reported experiencing challenges with brushing their teeth.

**Table 2:** Oral health status, behaviour and care utilization, n (%)

Self-reported oral health status	
Excellent	18 (9.5)
Very good	58 (30.7)
Good	40 (21.2)
Fair	60 (31.8)
Poor	13 (6.9)
Daily brushing frequency	
0	47 (24.8)
1	81 (43.0)
2 or more	61 (32.2)
Daily brushing supervision	
Independent	144 (76.0)
With supervision	11 (5.8)
With assistance	34 (18.2)
Dental visit in the last 24 months	
Yes	161 (85.1)
No	28 (14.9)
Last dental care provider	
General dentist	100 (52.9)
Pediatric dentist	77 (40.5)
Other	12 (6.6)
Preventive dental visit in the last 12 months	
0	77 (40.5)
1	70 (37.2)
2 or greater	42 (22.3)
Behaviour management	
None	61 (32.2)
Protective stabilization	23 (12.4)
Sedation (oral)	11 (5.8)
General anesthesia	92 (48.8)
Other	2 (0.8)
Unmet dental needs	
Yes	88 (46.6)
No	81 (42.9)
Unsure	20 (10.6)
Difficulty accessing dental care	
Yes	103 (54.5)
No	80 (42.3)
Unsure	6 (3.2)
Previous dental treatment	
Examination	180 (95.0)
Radiographs	170 (90.1)
Fluoride	153 (81.0)
Cleaning/prophylaxis	150 (79.3)
Scaling	37 (19.8)
Sealant	84 (44.6)
Filling	152 (80.2)
Extraction	128 (67.8)
SSC	95 (50.4)
Root canal therapy	75 (39.7)
Orthodontic	22 (11.6)

Table 3 and Table 4 show the association of different demographic variables and self-reported difficulty accessing oral health care for their child. For the univariable analysis (Table 3), annual family income, caregiver education level, primary residence location, and insurance status had a significant association with difficulty accessing oral health care. The multivariate analysis (Table 4) demonstrates that individuals with a higher education (post-graduate/doctoral) (OR= 0.68; 95% CI 0.54 – 0.91) or an income greater than $75,000 (OR=0.78; 95% CI 0.68 – 0.95) were less likely to report difficulty accessing care. Individuals who reported a high school education (OR=1.23; 95% CI 1.03 – 1.38); or depended on public insurance (OR=1.33; 95% CI 1.24 – 1.42) or out-of-pocket payments (OR=1.37, 95% CI 1.20 – 1.46); or lived in rural (OR=1.19, 95% CI 1.07 – 1.26) or remote (OR=1.23; 95% CI=1.12 – 1.31) locations were more likely to report having difficulty in accessing oral health care.

**Table 3:** Analysis of reported difficulty in accessing oral health care

Independent variables	Difficulty accessing dental care, n (%)		X^**2**^ Value	df	***p***-value
	Yes	No			
Child’s gender					
Male	60 (58.3)	38 (47.5)	4.27	1	0.15^Ŧ^
Female	43 (41.7)	42 (52.5)			
Caregiver status					
Biological parent	30 (29.1)	27 (33.8)	1.84	3	0.61^¥^
Relative	33 (32.0)	22 (27.5)			
Adoptive parent	36 (35.0)	30 (37.5)			
Foster parent	4 (3.9)	1 (1.3)			
Caregiver highest education					
Less than high school	10 (9.7)	13 (16.3)	9.34	3	0.03^*¥^
High school or equivalent	49 (47.6)	30 (37.5)			
Post-secondary	39 (37.9)	24 (30.0)			
Post-graduate/Doctoral	5 (4.9)	13 (16.3)			
Family income					
Less than $20,000	25 (24.3)	2 (2.5)	43.95	3	0.00^*¥^
$20,000 to $49,999	55 (53.4)	25 (31.3)			
$50,000 to $74,999	21 (20.4)	37 (46.3)			
Greater than $75,000	2 (1.9)	16 (20.0)			
Insurance status					
Private insurance	3 (2.9)	58 (72.5)	91.17	2	0.00^*¥^
Public insurance	89 (86.4)	22 (27.5)			
Out-of-pocket payments	11 (10.7)	0 (0.0)			
Primary residence					
Urban	54 5 (2.4)	48 (60.0)	3.47	2	0.02^*¥^
Rural	30 (29.1)	28 (35.0)			
Remote	19 (18.4)	4 (5.0)			

^*^Significance at 0.05 level; ^Ŧ^Chi-squared test; ^¥^ Fisher’s exact test

**Table 4:** Logistic regression model examining the reported difficulty in accessing oral health care

Independent variables	Difficulty accessing dental care, n (%)		Odds Ratio (95% Confidence Interval)	***p***-value
	Yes	No		
Caregiver highest education				
Less than high school	10 (9.7)	13 (16.3)	1	0.003
High school or equivalent	49 (47.6)	30 (37.5)	1.23 (1.03 – 1.38)	
Post-secondary	39 (37.9)	24 (30.0)	1.05 (0.96 – 1.21)	
Post-graduate/Doctoral	5 (4.9)	13 (16.3)	0.68 (0.54 – 0.91)	
Family income				
Less than $20,000	25 (24.3)	2 (2.5)	1	0.019
$20,000 to $49,999	55 (53.4)	25 (31.3)	1.18 (0.98 – 1.33)	
$50,000 to $74,999	21 (20.4)	37 (46.3)	0.89 (0.79 – 1.09)	
Greater than $75,000	2 (1.9)	16 (20.0)	0.78 (0.68 – 0.95)	
Insurance status				
Private insurance	3 (2.9)	58 (72.5)	1	0.030
Public insurance	89 (86.4)	22 (27.5)	1.33 (1.24 – 1.42)	
Out-of-pocket payments	11 (10.7)	0 (0.0)	1.37 (1.20 – 1.46)	
Primary residence				
Urban	54 5 (2.4)	48 (60.0)	1	0.012
Rural	30 (29.1)	28 (35.0)	1.19 (1.07 – 1.26)	
Remote	19 (18.4)	4 (5.0)	1.23 (1.12 – 1.31)	
Significance at 0.05 level				

Caregiver responses related to barriers to accessing oral health care are reported in Table 5. In our sample, 43% of caregivers report that they have experienced at least some frustration when trying to obtain oral health care for their child. Cost (63%), location of a dentist who would treat their child (55%), and scheduling (48%) were the most frequently cited external barriers to care. Common personal barriers to seeking out care were the child’s anticipated behaviour (78%), caregiver anxiety (60%), lack of perceived need (48%), and other health care priorities (40%). Regarding interpersonal barriers, 45% of caregivers reported that they had difficulties finding a dentist who was capable to treat their child without a referral, while 35% believed that their dentist did not have adequate knowledge related to their child’s condition and needs. Finally, 22% reported that they felt some form of discrimination or disrespect during their last appointment, while 35% believed that their dentist did not spend enough time with their child.

**Table 5:** Self-reported barriers to oral health care, n(%)

Question item	Never	Occasionally/Sometimes	Always
**External (environmental)**			
Office location too far away	85 (45)	81 (43)	23 (12)
Poor parking facilities	104 (55)	68 (36)	17 (9)
Transportation not available	89 (47)	76 (40)	24 (13)
Inconvenient appointment time	98 (52)	53 (28)	38 (20)
Wait time for appointment too long	104 (55)	68 (36)	17 (9)
Challenges with taking time off of work	117 (62)	57 (30)	15 (8)
Cost/financial difficulty	70 (37)	74 (39)	45 (24)
Insurance plan not accepted	77 (41)	76 (40)	36 (19)
**Personal**			
Prioritizing other health care needs	91 (48)	62 (32)	36 (19)
Child’s behaviour/cooperation	62 (33)	72 (38)	55 (29)
Child’s ability to communicate	64 (34)	79 (42)	45 (24)
Child’s anxiety towards dental treatment	45 (24)	76 (40)	68 (36)
Caregiver’s anxiety towards dental treatment	100 (53)	72 (38)	17 (9)
Caregiver stress associated with FASD	55 (29)	81 (43)	53 (28)
Child’s FASD will complicate dental care	83 (44)	68 (36)	38 (20)
Child only has baby teeth that will fall out	129 (68)	49 (26)	11 (6)
**Interpersonal**			
Inability to find a pediatric specialist	72 (38)	79 (42)	38 (20)
Inability to find a dentist willing to treat my child	112 (59)	45 (24)	32 (17)
Practitioner knowledge about FASD	66 (35)	85 (45)	38 (20)
Office staff knowledge about FASD	64 (34)	79 (42)	46 (24)
Dentist/staff do not listen to my concerns	89 (47)	76 (40)	24 (13)
Dentist/staff are disrespectful/discriminatory	115 (61)	64 (34)	9 (5)
Dentist/staff do not spend enough time with my child	96 (51)	59 (31)	34 (18)

## Discussion

This research examines the use of oral health care services for children diagnosed with FASD and barriers to accessing oral health care from the perspective of their parents/caregivers. While the majority of children (85%) had seen a dentist within the last 24 months, the percentage is lower than the national average of 91% reported in the Canadian Health Measures Survey for children between the ages of 6 to 11 [17]. Almost half (43%) of caregivers reported some barriers to accessing oral health care. We also found that socioeconomic factors such as family income, caregiver education level, primary residence location and insurance status had a significant association with difficulty accessing oral health care for this sample population.

The social determinants of health, which are shaped by the distribution of money, power, and resources, describe the conditions in which people are born, grow, live, work and age [18]. These non-medical factors influence health outcomes and inequities as the lower the socioeconomic status, the worse the oral health, which is consistent with our findings. Out of the factors we assessed, including income and education, half or more of our participants were on the lower end of the spectrum for these variables, which was associated with additional barriers to accessing care. When considering early childhood development, only about 30% of the caregiver participants were biological parents of their children, and 40% of caregivers were adoptive or foster parents.

The relationship between poverty and FASD are well established, with lower socioeconomic group generally having higher rates of FASD [19]. Income and insurance are also known risk factors for poor oral health outcomes [17, 20–22]. Canadians who report a lower annual income were found to have higher rates of dental caries, lower utilization rates for oral health care services, and were more likely to postpone or decline recommended care due to costs [17]. In our sample population, 63% of caregivers reported cost as barrier to accessing care. While many caregivers had access to public insurance, it is important to note that not all oral health care services (e.g. sedation or general anesthesia) are eligible benefits in public plans. Additionally, cost may refer to both the direct costs for treatment and indirect costs such as transportation, parking, as well as loss of employment income if caregivers had to take time off work to arrange for their child’s care.

Additional external environmental factors which have been described for children with developmental disabilities include structural barriers, transportation difficulties and inadequate facilities [14, 15]. In our study, caregivers frequently cited location (55%) and scheduling (48%) as barriers. Only about half of our participants lived in an urban location, so many caregivers would have to travel far distances to reach the nearest dental clinic. Some patients with developmental disabilities may need special modifications, such as protective stabilization, sedation, or general anesthesia, that are not available in all dental clinics [14]. Almost half of the children in our study required general anesthesia for some dental treatment, which would also require access to specific facilities.

Children diagnosed with FASD may present with additional medical, physical, cognitive, communication, and behavioral issues that make routine treatment in a dental office challenging Access for oral examinations and procedures may be difficult if patients have hypersensitivity, poor oral tolerance, and limited mouth opening [2, 3]. Communication difficulties or social relatedness impairments may limit a patient’s ability to describe pain or articulate symptoms as well as cooperate with oral health care professionals, which may impede diagnosis and treatment [14]. Anticipated behavioral issues of the child at the dental visit was a frequently reported barrier (78% of caregivers). Dental anxiety and/or fear, of either patient or caregiver, may lead to avoidance or appointment cancellations. Approximately 60% of caregivers reported that their own dental anxiety was a barrier to accessing oral health care for their child. Patients or caregivers may perceive a lack of need for oral health care, as did 48% of caregivers in our study, and some may prioritize other medical needs, as did 40% of our participants. Our findings were also consistent with previous findings demonstrating that internal personal factors were more likely to represent barriers to oral health care than external environmental factors [14].

Lastly, barriers to accessing care can be interpersonal, referring to the relationships between oral health care professionals, patients and their caregivers. Dentists and other oral health care staff may be unable, uncomfortable, or unwilling to treat patients with special health care needs due to inadequate training, knowledge, and experience [15]. Previous research showed that 56% of caregivers had experienced dental offices refusing or being unable to treat their child with special health care needs [23]. Almost half of the caregivers in our study reported having difficulties finding oral health care professionals who were capable of treating their child without a referral. Additionally, dentists in private practice, including both general and pediatric specialists, failed or lacked knowledge and capacity to engage children with disabilities [23]. Our findings are consistent with this research, as we report that 35% of caregivers thought that their dentist lacked adequate knowledge about their child’s FASD condition and needs, and 22% reported experiencing some discrimination or disrespect during their child’s dental visit.

The strengths of this study include that it is one of the first to explore access to oral health care for children diagnosed with FASD and our sample size allowed us to make some inferences about this population. Questions in our survey instrument adapted from other surveys allowed comparison of our results to research involving children with other developmental disabilities. However, it is also important to note the limitations of this study. Due to the cross-sectional design, this study cannot determine temporality or causation. There may be sample bias, as those with the most needs may be more inclined to participate. Participants may also have recall bias, so they may not have accurately remembered their dental visits or perceptions. Additionally, the generalization of our findings are limited as we did not include a control group. Nonetheless, the findings from this research offer insight into the perceived barriers to oral health care for children diagnosed with FASD and will stimulate further research.

## Conclusion

Our findings indicate that children with FASD experience many different barriers to accessing timely oral health care. Social determinants of health were significant variables that increased likelihood of access-to-care barriers. Similar to other vulnerable populations, the cost of oral health care and location of dental clinics are notable barriers. However, we also reveal challenges associated to behaviour specific to children with FASD; thus, oral health care providers’ ability to assess and manage this behaviour is a noteworthy area. Furthermore, the possibility of any stigma associated with FASD affecting oral health care is an area requiring further investigation. Recognizing the challenges related to access to oral health care will help clinicians, public health professionals, and policymakers adjust current care practices as well as develop appropriate programs and resources to break down barriers and improve the oral health status of children with FASD.

## Data Availability

The datasets used and/or analyzed during the current study are available from the corresponding author on reasonable request.
